# GestureMoRo: an algorithm for autonomous mobile robot teleoperation based on gesture recognition

**DOI:** 10.1038/s41598-024-54488-w

**Published:** 2024-03-14

**Authors:** Lei Chen, Chunxu Li, Ashraf Fahmy, Johann Sienz

**Affiliations:** 1 Department of Machenical Engineering, Faculty of Science and Engineering, Swansea University, Swansea, SA1 8EN UK; 2 College of Mechanical and Electrical Engineering, Hohai University, Changzhou, 213200 China

**Keywords:** GestureMoRo, Gesture recognition, LeapMotion, Mobile robot, Teleoperation, Gaussian filter, Mechanical engineering, Computer science

## Abstract

Gestures are a common way people communicate. Gesture-based teleoperation control systems tend to be simple to operate and suitable for most people’s daily use. This paper employed a LeapMotion sensor to develop a mobile robot control system based on gesture recognition, which mainly established connections through a client/server structure. The principles of gesture recognition in the system were studied and the relevant self-investigated algorithms—GestureMoRo, for the association between gestures and mobile robots were designed. Moreover, in order to avoid the unstably fluctuated movement of the mobile robot caused by palm shaking, the Gaussian filter algorithm was used to smooth and denoise the collected gesture data, which effectively improved the robustness and stability of the mobile robot’s locomotion. Finally, the teleoperation control strategy of the gesture to the WATER2 mobile robot was realized, and the effectiveness and practicability of the designed system were verified through multiple experiments.

## Introduction

With the development of science and technology, robots no longer only exist in factories, but are also more common in life^1^. In line with the concept of people-oriented, in order to better control the robot, the way of human–computer interaction is also constantly enriched. Perhaps the most common human–computer interaction methods we use in life are semantic interaction and gesture interaction. Semantic interaction allows us to operate robots or other digital devices through voice commands^2^. However, there are many languages in the world, and the same language has different dialects and accents. Therefore, the prerequisite for realizing semantic interaction that is applicable to everyone tends to require a huge database. In addition, the tone and context of the user’s speech is also supposed to be considered, which is relatively difficult and costly. On the contrary, all ethnic groups in the world have the same hands, so the applicability of gesture interaction is indistinguishable for each ethnic group, and gesture interaction is easy to operate^3^.

Gestures are one of the most important ways of expression in daily communication. With the development of robots in the medical field, gesture interaction is increasingly helpful to disabled people. For example, it is entirely possible for people with disabilities to abandon the traditional way of controlling the movement of wheelchairs and use non-contact gestures to control wheelchairs. Therefore, gestures are a good choice as a way of human–computer interaction, and there is a few technical difficulties in terms of user experience. Predecessors had also conducted plenty of research works on gesture recognition. In the literature^4^, Kinect sensor was used for gesture recognition to control the movement of mobile robots. In this paper, the Kinect sensor could recognize the movement of the operator’s arm, and the range of recognition was also relatively large. However, it mainly used skeleton analysis and cannot identify the specific details of the operator’s hands. In the paper^5^, Sun et al. proposed a new method to use the depth information of neighboring pixels to restore missing depth data. This method overcomes the shortcomings of traditional methods that easily produce artifacts or blurring at the edges of objects., and has good results if applied to a gesture recognition system to identify the shape of the finger. In the paper^6^, the author considered that the Kinect sensor may cause gesture recognition errors because of the similarity of two interactive actions, and that the system may recognize people’s unconscious body movements as positive gestures, so this paper proposed to combine Kinect with visual and deep learning algorithms to effectively improve the identification of conflict phenomena and determine unconscious actions. A RGB-based deep learning gesture recognition method was studied in the research work^7^, in which they shared a dataset of real and synthetic control gestures, and proposed a gesture recognition solution based on the I3D model. They demonstrated that synthetic data are crucial in overcoming the challenges of collecting large amounts of annotated data on human subjects. Of course, some researchers also used radar for gesture recognition. In the literature^8^, a new type of low-complexity lidar gesture recognition system was proposed. The system was divided into a pose estimation module and a gesture classifier, thereby reducing the dimensionality of the input while also easing the computational burden on mobile robots with relatively limited computing power. Literature^9^ was another example of gesture recognition using radar. In this paper, the researchers proposed a robust gesture recognition method based on dual-Doppler radar, combined with improved dynamic time warping (DTW) algorithms to classify gestures between humans and radar, a collaborative robot control system was also developed.

In order to obtain a better experience and more precise operation in the process of controlling the robot with gestures, some researchers used multi-source data fusion for gesture recognition. In the research work^10^, researchers unified the operation, visual/force feedback areas, and introduced voice interaction to improve the accuracy of teleoperation and the naturalness of interaction, thereby achieving an immersive and natural interaction environment. In order to enhance the user experience in gesture control of the robot with respect to the immersiveness, operation convenience, and control accuracy, in^11^, researchers developed an application of augmented reality (AR) in robot control and adjustment. In their research, they used the Kalman filter (KF) algorithm to fuse the gesture coordinate data obtained by the LeapMotion sensor and the movement speed of the gesture in the three-dimensional space obtained by the Kinect V2 camera, thereby better realizing the teleoperation of the Baxter robot with gestures. In addition, in the literature^12^, researchers designed an adaptive teleoperation system for the DLR-HIT II manipulator based on neural networks. In this system, they corresponded the joints of the operator’s hand with the joints of the robot’s hand based on the LeapMotion device, so as to realize the teleoperation control of the manipulator. Most of the above-mentioned gesture control robot systems added many auxiliary devices in order to improve the accuracy or function of the system, ignoring the convenience of user use. The teleoperation control system developed in our paper realized the convenience and safety of user operation while ensuring high-precision control of the robot.

The positioning of the paper is to provide the prototype of a new smart wheelchair control method for people with disabilities, and in this method, the control of any movement mode of the smart wheelchair can be achieved with simple gestures. The main goal is to reduce the burden of controlling smart wheelchairs for people with disabilities. Solly et al.^13^ proposed a gesture control system based on a glove controller to control a mobile robot. They required the operator to wear a professional glove controller to control the mobile robot. Although control accuracy was guaranteed, they also clearly pointed out that this control method required higher movement and control abilities of the user, so it was not suitable for everyone, especially the elderly. Yamashita et al.^14^ proposed a mobile robot control method based on fingertip gesture recognition, and this method could be integrated with the face recognition system to improve the interactivity of the mobile robot controlled by fingertip gestures. However, the fingertip gesture recognition was performed through a glasses-type wearable camera. Therefore, the gesture was often misrecognized due to the noise generated by the shaking of the user’s head during use. The average accuracy of the gesture recognition was 84%, which was still relatively low. In addition, fingertip gesture recognition required the operator’s fingers to be flexible and constantly changing finger postures. However, our research can easily control the mobile robot through simple gestures of the palm. Wang et al.^15^ designed an intelligent mobile robot control system based on myoelectric signals to achieve remote control of the car through the control of myoelectric signals. However, in the system they designed, it was difficult to identify the hand muscles when the operator opens his hand, which requires the operator to always keep his hand tight, which brings difficulties to the operator. Chamorro et al.^8^ proposed a novel low-complexity lidar gesture recognition system for robust control of gesture changes in mobile robots. Its gesture classification was completed using a long short-term memory network and used a series of estimated body postures as input to predict gestures, so its control accuracy was high. However, in their system, 18 kinds of gestures were used to achieve remote control of the mobile robot. The gestures were relatively complex, and the set gestures included the entire arm. Therefore, the operator had a large range of movement during the remote control process, and the changes in control instructions were relatively slow.

Therefore, this paper possesses the following novelties compared with the studies mentioned above: first, the teleoperation system designed in this paper does not require the operator to wear any equipment, which reduces the operator’s burden. Secondly, the gesture recognition system developed by this research only requires the operator to naturally relax and open his hand over the LeapMotion sensor, and the operator only needs to control the forward and backward tilt angle and left and right roll angle of the palm to realize the speed control and direction control of the mobile robot. Thirdly, the three-dimensional coordinate values obtained from the LeapMotion in this paper were not directly mapped to the world coordinate system, but mapped separately, which effectively reduced the cumulative error of direct mapping between two three-dimensional coordinates. Therefore, it is suitable for most people and requires no professional knowledge to get started. This research is suitable for smart wheelchairs and provides a contactless and labor-saving strategy for disabled people to control smart wheelchairs.

In addition, achieving continuous, stable, and high-precision control of mobile robots is crucial for the widespread application of mobile robots^16^. For example, in multi-objective control, due to the existence of dynamic instability, the discontinuous solution of the optimization algorithm may cause chatter, which will greatly affect the stability of the robot^17,18^. Also in cruise control, switching control inputs may cause user discomfort and safety issues^19^. Therefore, a large amount of research had been devoted to solving how to improve the continuity, stability and control accuracy of mobile robots. In recent years, with the development of reinforcement learning, many researchers had used reinforcement learning to develop computer models of robot control decisions, enabling robots to autonomously acquire skills to perform complex tasks, such as robot manipulation, locomotion, and navigation^20–24^, thereby achieving autonomous and continuous control of the robot. However, this method was not efficient. It needed dense rewards to keep the actions under supervision at all times, so as to improve the learning efficiency. There were also researchers who used dynamic control to achieve precise dynamic adjustment of the mobile robot chassis and improve tracking performance^25–27^. In addition, there are often unanticipated factors such as actuator failure and input saturation in mobile robot systems, so it is difficult to achieve high-precision control of mobile robots using traditional control methods of robot models. In recent years, based on sliding mode^28–32^, robust adaptive^33–37^, fuzzy logic^38–40^ and other technologies, various advanced control strategies had been developed to solve various problems in practical systems. Among them, in terms of actuator failure, in the literatures^41,42^, an iterative learning controller and a distributed local controller were constructed respectively to ensure trajectory tracking and satisfactory transient performance of two driving wheel mobile robots in the case of deviation actuator failure. In these studies, robust asymptotic tracking and transient response were highly considered in actuator faults and system uncertainties. The stability, continuity and high-precision control of the mobile robot in our paper were not only realized by the characteristics of the WATER2 mobile robot having two driving wheels and four universal wheels, but also mainly relied on the mapping algorithm from the gesture to the mobile robot designed by ourselves, the high precision of LeapMotion itself and the Gaussian filtering algorithm.

The paper mainly realized the teleoperation control of WATER2 mobile robot through the method of dynamic gesture recognition based on LeapMotion device. The content of the paper was distributed as follows: The first section introduced the research background and significance of this paper. The second section introduced the preparation work of this paper, including the hardware, software and workstation used in the experiment. The third section mainly introduced the methodology of this paper, including the kinematic modeling of mobile robots, the design of LeapMotion control flow, the design of the association algorithm between gestures and mobile robots, and the principle of Gaussian filtering algorithm. The fourth section was the experimental part of this paper, including the construction of the experimental platform, the design of the experimental plan, and the analysis of the experimental results. The fifth section was the conclusion and prospect of this paper. Compared with traditional teleoperation methods^43,44^, the innovations and unique contributions of this paper can be succinctly summarized as follows:

1. The model coupling between the mobile robot and LeapMotion was realized. We integrated the palm position and the speed of the mobile robot to ensure high-precision control of the mobile robot.
2. Based on the LeapMotion sensor, we designed a correlation algorithm between gestures and mobile robots, and named it the GestureMoRo algorithm.
3. The Gaussian filter algorithm was integrated into the control system we designed, and the data collected by LeapMotion were processed by Gaussian filter, thereby avoiding the unstable motion of the mobile robot caused by the shaking of the palm during gesture control.
4. Compared with other studies using LeapMotion sensors, the three-dimensional coordinate values obtained from the LeapMotion coordinate system in this paper were not directly mapped to the world coordinate system, but the three-dimensional coordinates were mapped separately, thus effectively reducing the cumulative error of direct mapping between two three-dimensional coordinates.

## Preliminaries

**Figure 1:**
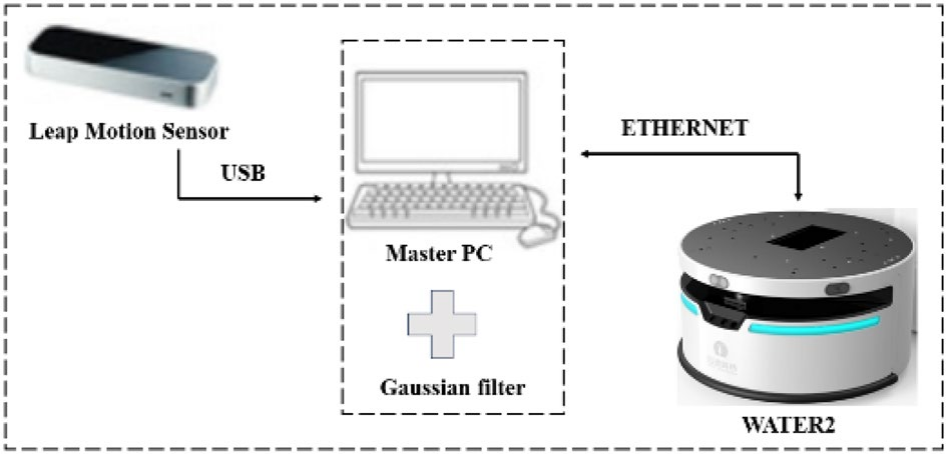
Control system structure.

The hardware used to implement the human–computer interaction system in this paper included LeapMotion, laptop computers and mobile robots. The structure of the control system was shown in Fig. 1.

### LeapMotion

**Figure 2:**
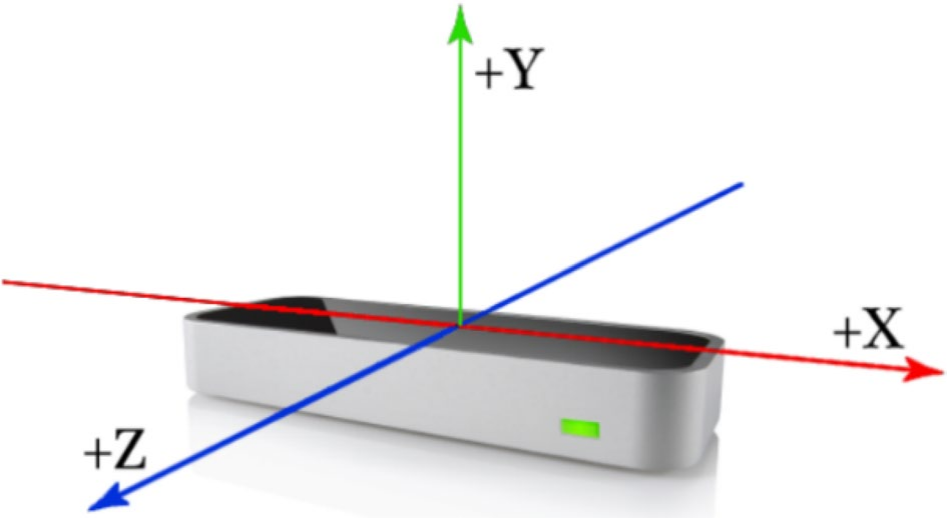
LeapMotion coordinate system.

**Figure 3:**
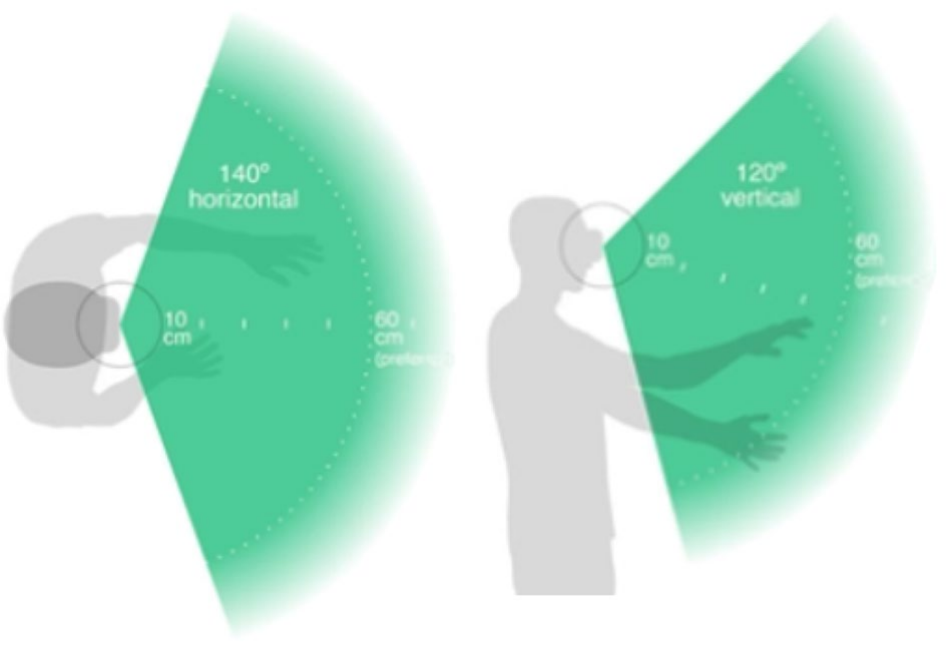
LeapMotion tracking range.

The size of the LeapMotion sensor is 80 mm long × 30 mm wide × 11.3 mm high, as shown in Fig. 2. It employs a right-handed Cartesian coordinate system, with the origin positioned at the center atop the LeapMotion sensor^45^. Specifically, the x-axis and z-axis lie within the horizontal plane, with the x-axis aligned parallel to the device’s longer side, and the z-axis parallel to the shorter side. Meanwhile, the y-axis extends vertically upward, with positive values increasing upward, and the z-axis has positive values increasing toward the user. Its trackable range was shown in Fig. 3. The horizontal field of view is about 140°, the vertical field of view is about 120°, and the recognition range perpendicular to the LeapMotion surface is 25–600 mm^46^. The officially provided LeapMotion accuracy is 0.01 mm, but the actual accuracy may be 0.7 mm. The accuracy is still high, and 200 frames of hand data can be collected per second.

LeapMotion is equipped with two 120 frame rate gray-scale infrared cameras and three infrared LEDs. The infrared LEDs emit light that penetrates the filter layer and illuminates the target object for tracking. The camera receives the reflected infrared light. Then, the hand’s three-dimensional position is determined using binocular stereo vision imaging principles, enabling the creation of a precise three-dimensional hand model. At the same time, visible light cannot pass through the filter layer, shielding the complex background, thereby reducing the complexity of post-processing.

### WATER2 mobile robot

**Figure 4:**
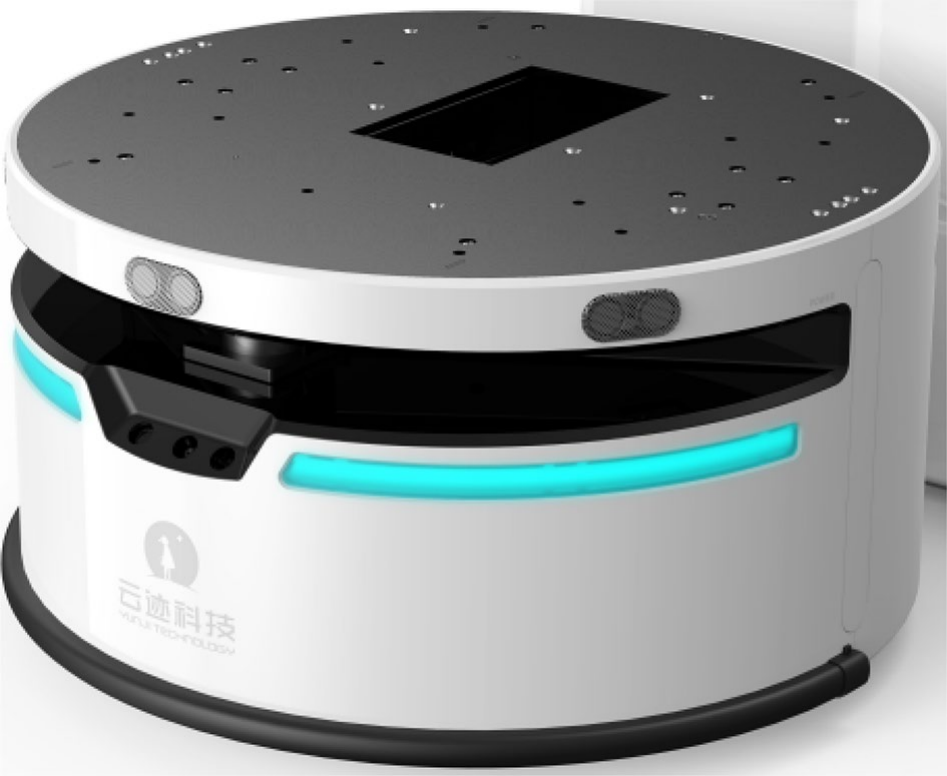
WATER2 mobile robot.

As shown in Fig. 4, the WATER2 mobile robot produced by Beijing Yunji Technology Co. Ltd. has a built-in wireless router, which can communicate with the PC through WIFI.

### Workstation

The PC used in this paper was a ThinkPad E580 laptop equipped with Windows 10 operating system. The development language used was python2.7. The compiler used was pycharm imported from anaconda, and python SDK V3 was installed.

## Methodology

### Kinematic modeling of mobile robots

**Figure 5:**
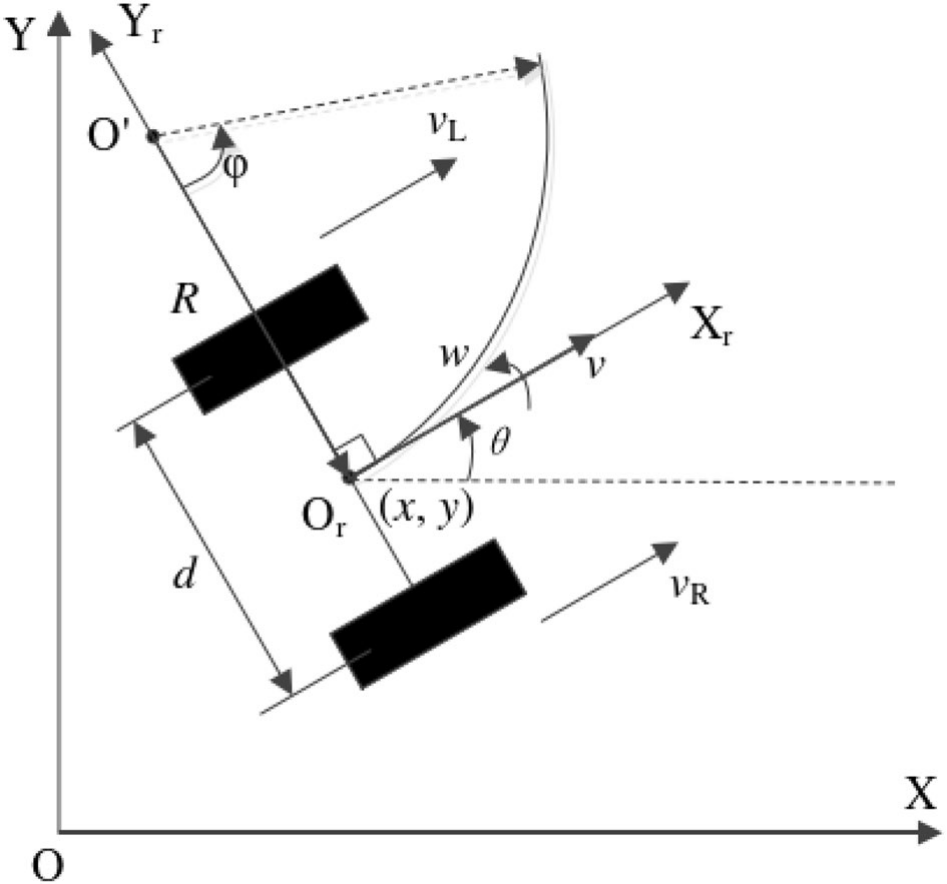
Differential drive mobile robot kinematics model.

The WATER2 mobile robot used in this paper has 6 wheels, including four symmetrically distributed universal wheels. Their main function is to increase the stability of the mobile robot during operation, but they do not have a driving effect; There are two symmetrically distributed driving wheels, whose main function is to drive the mobile robot to move through differential driving. Its motion coordinate system and simplified model were shown in Fig. 5.

In Fig. 5, we illustrated the kinematic model of a differential drive mobile robot within the two-dimensional world coordinate system, denoted as $OXY$. The mobile robot’s body coordinates, represented as $O_{r} X_{r} Y_{r}$, have their origin, $O_{r}$, precisely positioned at the midpoint of the two wheel axles—also serving as the robot’s center point. The forward direction of the robot is established as the positive x-axis ($X_{r}$), while the positive y-axis ($Y_{r}$) is determined following the right-hand rule. Consequently, the robot’s pose with respect to the world coordinate system, denoted as $OXY$, is precisely recorded as:

1 $$ q = \left[ {\begin{array}{*{20}c} x & y & \theta \\ \end{array} } \right]^{T} $$

where, $\left( {x,y} \right)$ represents the position coordinates of the mobile robot in relation to the world coordinate system $OXY$. Additionally, $\theta \in \left( { - \pi ,\pi } \right]$ signifies the angle between the mobile robot’s orientation and the positive X-axis of the world coordinate system, with counterclockwise direction regarded as positive.

Differential drive mobile robots complete steering by independently controlling the wheels on both sides of the robot. Assume that the instantaneous rotation center of the mobile robot is point $O^{\prime }$, its left wheel speed is $v_{L}$, and its right wheel speed is $v_{R}$. Then the instantaneous radius $R$ of the mobile robot’s instantaneous speed center $O^{\prime }$ is calculated as Eq. (2):

2 $$ R = \frac{v}{\omega } = \frac{{\left( {v_{L} + v_{R} } \right)/2}}{{\left( {v_{L} - v_{R} } \right)/d}} $$

where $v$ represents the linear velocity (m/s) of the mobile robot’s center, $\omega$ denots the angular velocity (rad/s) of the mobile robot, $v_{L}$ is as the speed of the left wheel of the mobile robot, $v_{R}$ indicates the speed of the right wheel, $d$ signifies the distance between the two drive wheels of the mobile robot, with units measured in meters (m).

The motion of the mobile robot at any moment can be decomposed into the linear velocity ($v$) of the center of the mobile robot and the angular velocity ($\omega$) rotating around the instantaneous center $O^{\prime }$. Its kinematic positive solution equation is shown in formula (3):

3 $$ \left[ {\begin{array}{*{20}c} v \\ \omega \\ \end{array} } \right] = \left[ {\begin{array}{*{20}c} \frac{r}{2} & \frac{r}{2} \\ { - \frac{r}{d}} & \frac{r}{d} \\ \end{array} } \right]\left( {\begin{array}{*{20}c} {\omega_{L} } \\ {\omega_{R} } \\ \end{array} } \right) = J*\left[ {\begin{array}{*{20}c} {\omega_{L} } \\ {\omega_{R} } \\ \end{array} } \right] $$

where $v$ representes the linear velocity (m/s) of the mobile robot’s center, $\omega$ denotes the angular velocity (rad/s) of the mobile robot, $\omega_{L}$ and $\omega_{R}$ are angular velocities (rad/s) of the left and right driving wheels of the mobile robot, $r$ is the radius of the driving wheel on the mobile robot. $d$ is the distance between the two driving wheels of the mobile robot.

The inverse kinematic solution of the mobile robot was obtained from Eq. (3), as shown in Eq. (4):

4 $$ \left[ {\begin{array}{*{20}c} {\omega_{L} } \\ {\omega_{R} } \\ \end{array} } \right] = \left[ {\begin{array}{*{20}c} r & { - \frac{dr}{2}} \\ r & \frac{dr}{2} \\ \end{array} } \right]\left[ {\begin{array}{*{20}c} v \\ \omega \\ \end{array} } \right] = J^{ - 1} *\left[ {\begin{array}{*{20}c} v \\ \omega \\ \end{array} } \right] $$

Finally, the ($v,\omega$) values received from the host computer were employed to execute the inverse kinematics calculation using Eq. (4), yielding the projected angular velocities for the left and right drive motors. This process underpined the effective motion control of the robot.

### Control flow

As the LeapMotion controller tracks hands and fingers in its field of view, it provides updates in the form of data sets, or frames. Each frame object represents all tracked hands and details their properties at the current moment. The frame object is essentially the root of the LeapMotion data model.

First, we found the three files *Leap.py*, *LeapPython.pyd* and *Leap.dll* in the lib folder of the Python library in the Leap SDK, and save the three files to the same directory of the python source code program we developed. Just *import leap* in program.

Secondly, we realized the connection between the computer and the mobile robot through wifi wireless communication, and then realized the connection between the comput and the mobile robot on the program through the *socket* module. We used *controller* = *Leap.Controller()* to add the *Controller* object to the program. When creating the *Controller* object, It automatically connected to the LeapMotion service, and once a connection was established, tracking data would could be obtained from it using the *Controller.frame()* method. Then use *controller.add_listener(listener)* to let the sample *listener* receive events from the *controller*, so that it can receive data from the *controller* in real time, that is, information for each *Frame*.

In addition, since the duration of a single command of the WATER2 mobile robot used is 0.5 s, the input frequency can be greater than 2 Hz. Therefore, in order to make the movement of the mobile robot coherent during gesture control, this paper set 20 ms as a cycle to send commands.

### Algorithm design

This paper proposed an association algorithm between gestures and mobile robots based on LeapMotion, named GestureMoRo algorithm. Within this algorithm, we’ve translated gesture movements into two distinct components for controlling the mobile robot. The initial component involves the vertical displacement of the left hand’s palm concerning the LeapMotion sensor, coupled with the palm’s pitch angle, which correlates with the robot’s linear velocity. The second part is the roll angle of the left hand corresponding to the angular velocity of the mobile robot.

**Figure 6:**
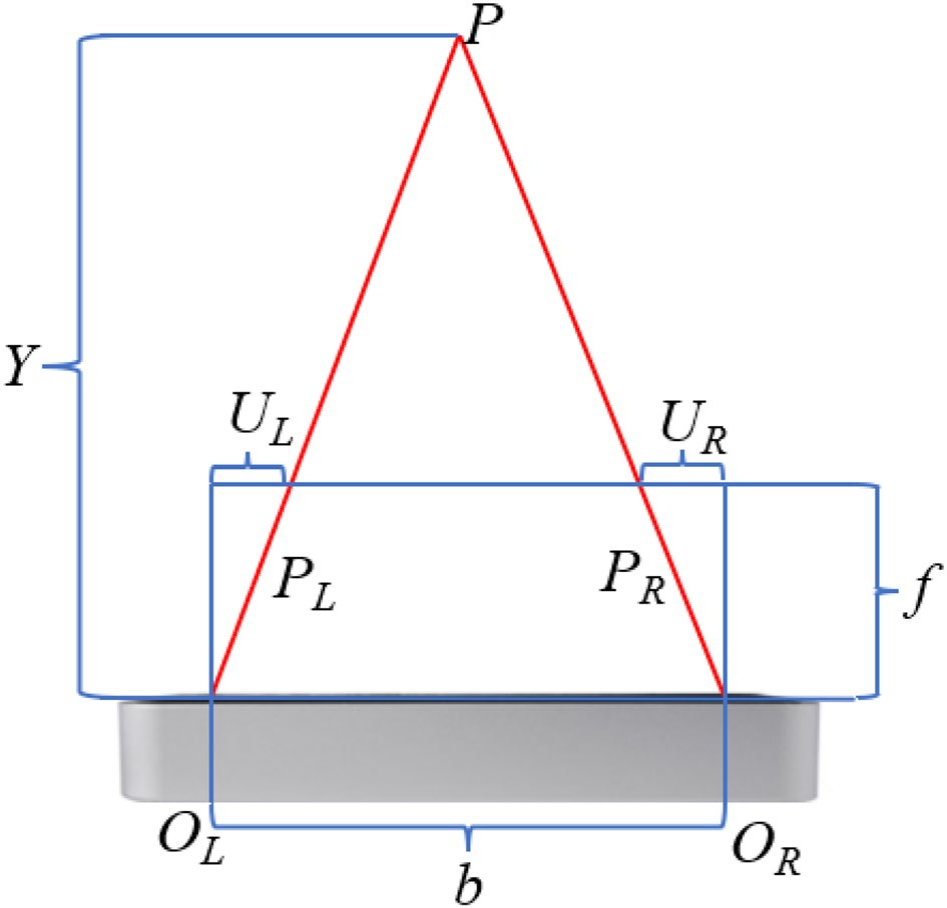
Binocular camera imaging principle, modified from^47^.

The vertical distance from the palm of the left hand to the LeapMotion sensor is the coordinate value of the palm of the hand on the Y-axis. This value was obtained by using the *hand.palm_position* attribute to obtain the position of the palm through the stereoscopic vision principle of the binocular camera. When the operator’s hand appeared in its working area, the binocular camera detects the target at the same time and calculated the target’s depth information based on the target’s parallax. The final palm information was the palm information processed by the parallax principle. The imaging principle of the binocular camera is shown in Fig. 6:

Among them, $P$ is the target point for detection, b is the aperture center separation distance between the two cameras, $U_{L}$ is the distance from the left aperture center to the left imaging point, $U_{R}$ is the distance from the right aperture center to the right imaging point. $y$ is the distance between the target point and the aperture center, $f$ is the focal length, and the parallax is $d$ = $U_{L}$ + $U_{R}$. The Z-axis is orthogonal to both the X and Y axes. The Z-coordinate for the left camera is denoted as $Z_{L}$, while for the right camera, it’s represented as $Z_{R}$.

Then calculated the x, y, and z values of point $P$. According to the similar triangle criterion, formula (5) could be obtained. Then formula (6) was derived from formula (5). Thus, the depth value $y$ and the values of $x$ and $z$ were obtained. In this way, the binocular camera could be used to obtain the depth coordinate data of the object being photographed.

5 $$ \left\{ {\begin{array}{*{20}l} {\left( {b - U_{L} - U_{R} } \right)/b = \left( {y - f} \right)/y} \hfill \\ {y/f = \left( {b - x} \right)/\left( {b - U_{R} } \right)} \hfill \\ {y/f = z/z_{L} = z/z_{R} } \hfill \\ \end{array} } \right. $$

6 $$ \left\{ {\begin{array}{*{20}l} {y = fb/\left( {U_{L} + U_{R} } \right) = fb/d} \hfill \\ {x = yU_{L} /f = \left( {b - yx_{R} } \right)/f} \hfill \\ {z = yz_{L} /f = yz_{R} /f} \hfill \\ \end{array} } \right. $$

where $b$ is the distance between the aperture centers of the two cameras, $U_{L}$ is the distance between the left aperture center and the left imaging point, $U_{R}$ is the distance between the right aperture center and the right imaging point, $y$ is the distance between the target point and the aperture center, $f$ is the focal length. $Z_{L}$ is the coordinate of the left camera on the Z axis, and $Z_{R}$ is the coordinate of the right camera on the Z axis.

In order to avoid the mobile robot moving due to unintentional shaking of the palm, it was necessary to consider the range of the palm position to trigger the movement command of the mobile robot during the program development process. Combined with the recognition range of the LeapMotion sensor in the vertical direction, which is 25–600 mm, after many experimental tests, we set the linear speed mapped to the mobile robot to be 0 when the vertical distance $y$ between the palm and the sensor was less than 200 mm. When the distance $y$ was greater than 400 mm and less than 600 mm, the linear speed mapped to the mobile robot was 0.5 m/s. When the vertical distance y was within the range of 200 mm-400 mm, it was mapped to the linear speed $v$ of the mobile robot through formula (7).

7 $$ v = \left( {y - 200} \right)/400 $$

where $v$ is the linear speed of the mobile robot, and $y$ is the vertical distance from the palm to the LeapMotion.

**Figure 7:**
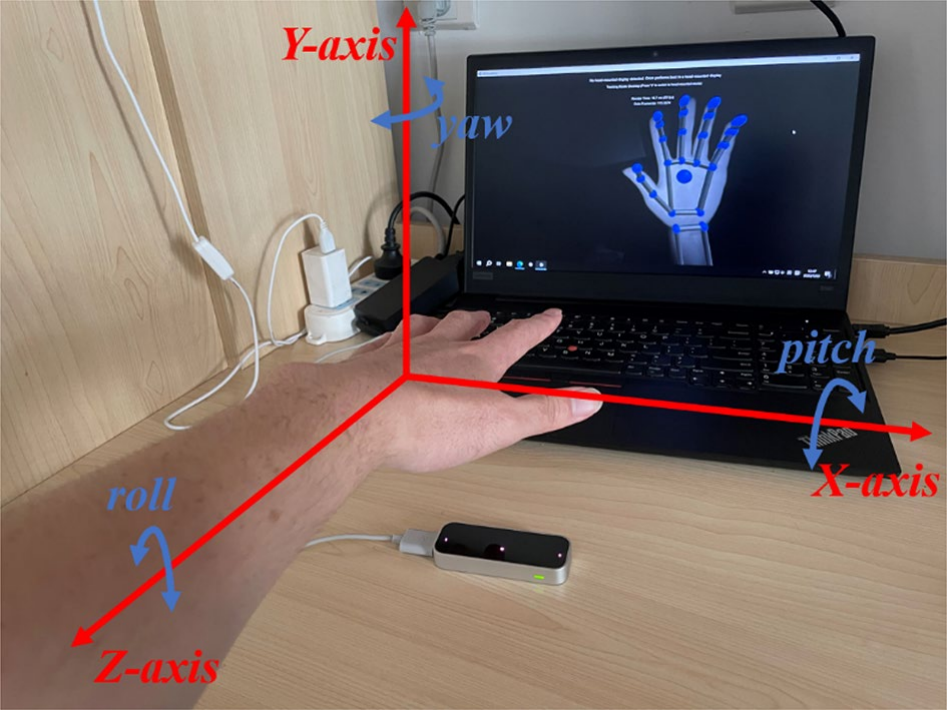
Schematic diagram of gesture deflection.

The schematic diagram of the gesture deflection angle of the LeapMotion sensor is shown in Fig. 7.

The pitch angle and roll angle of the left palm were obtained through the two properties of *hand.direction.pitch* and *hand.palm_normal.roll* respectively. The direction in the *hand.direction.pitch* attribute refers to the direction from the palm position to the finger. We can use the palm direction vector to calculate the pitch angle and yaw angle of the palm relative to the horizontal plane. The *palm_normal* in the *hand.palm_normal.roll* attribute refers to the direction of the palm normal vector, which can be used to calculate the rolling angle of the palm relative to the horizontal plane.

As we can see from the Fig. 7, the pitch angle of the left hand refers to the angle of rotation of the palm around the X-axis. The pitch is a negative value when the palm is buckled inward, and the pitch is a positive value when the palm is tilted upward. For safety reasons and to prevent the operator from accidentally triggering the mobile robot’s retreat command during teleoperation, after many tests, the program in this paper set that when the left hand buckles inward with a pitch less than − 5°, the robot’s linear speed passed through the formula (8) correspondingly change to a negative value, so as to realize the backward action.

8 $$ v = - v $$

The corresponding relationship between the linear speed of the mobile robot and gestures in this algorithm is shown in Eq. (9):

9 $$ v = \left\{ {\begin{array}{*{20}l} {0,} \hfill & {y\left\langle {200\;or\;y} \right\rangle 600} \hfill \\ {(y - 200)/400,} \hfill & {200 \le y\left\langle {400\;and\;pitch} \right\rangle - 5^\circ } \hfill \\ { - (y - 200)/400,} \hfill & {200 \le y < 400\;and\;pitch \le - 5^\circ } \hfill \\ { 0.5,} \hfill & {400 \le y\left\langle {600\;and\;pitch} \right\rangle - 5^\circ } \hfill \\ { - 0.5,} \hfill & {400 \le y \le 600\;and\;pitch \le - 5^\circ } \hfill \\ \end{array} } \right. $$

where $v$(m/s) is the linear speed of the mobile robot, and $y$(mm) is the vertical distance from the palm to the LeapMotion, and $pitch$ is the angle of rotation of the palm around the X-axis.

The roll angle of the left hand refers to the angle of rotation of the palm around the Z-axis. When the palm is flipped to the left, the roll angle is a positive value, and when the palm is flipped to the right, the roll angle is a negative value. For safety reasons and to prevent the operator from accidentally triggering the mobile robot’s turn command during teleoperation, after many tests, the program in this paper was set to trigger the mobile robot’s left turn command when the roll angle was greater than 20°. When flipping to the right, the command to turn the mobile robot to the right was triggered when the roll angle was less than − 20°.

The corresponding relationship between the specific palm roll angle of the algorithm we developed and the angular velocity of the mobile robot is as shown in Eq. (10):

10 $$ \omega = \left\{ {\begin{array}{*{20}l} { - 1,} \hfill & {roll < - 60^\circ } \hfill \\ {(roll + 20)/40,} \hfill & { - 60^\circ < roll < - 20^\circ } \hfill \\ {0,} \hfill & { - 20^\circ < roll < 20^\circ } \hfill \\ {(roll - 20)/40,} \hfill & {20^\circ < roll < 60^\circ } \hfill \\ {1,} \hfill & {roll > 60^\circ } \hfill \\ \end{array} } \right. $$

where $\omega$ is the angular velocity of the mobile robot, and $roll$ is the flip angle of the left hand.

### Filtering

In the process of controlling a mobile robot, the palm of the hand would inevitably shake unintentionally or shake violently, causing the mobile robot to stop and move suddenly during the movement, which greatly reduced the stability and robustness of the mobile robot. At the same time, there were also security risks. Therefore, in order to solve the problem of gesture jitter during control, it is necessary to perform anti-shake filtering on the collected palm data, and then send the processed data to the mobile robot.

In order to obtain better filtering effects, this paper used two filtering algorithms, exponential weighted moving average filtering and Gaussian filtering, to compare the filtering and denoising effects, thereby achieving effective smoothing and denoising effects on the data. Both of these filters performed anti-shake filtering on the speed v calculated from the distance between the center of the palm and the LeapMotion sensor in the y-axis direction.

#### Exponentially weighted moving average filter

Exponentially weighted moving average filtering mainly used the weighted average method to achieve smooth denoising effect on one-dimensional data. This weighting method had a certain smoothing effect on the impact of noise on the data. When the speed array to be processed was $\left( {v_{1} ,v_{2} ,v_{3} , \ldots v_{k} } \right)$, its mathematical expression was as shown in Eq. (11):

11 $$ S_{k} = \left\{ {\begin{array}{*{20}l} {v_{1} ,} \hfill & {k = 1} \hfill \\ {\alpha *v_{k} + \left( {1 - \alpha } \right)*v_{k - 1} ,} \hfill & {k > 1} \hfill \\ \end{array} } \right. $$

where, $v_{k}$ was the kth speed value in the speed array to be processed; α was the weight parameter, usually also called the attenuation factor; ${S}_{k}$ was the kth speed value after exponential weighted moving average filtering.

The main purpose of using exponentially weighted moving average filtering in this paper was to smooth and denoise the velocity. The impact of the previous value on the latter value in the velocity array should be taken into account. After many tests, the α value of this paper was 0.08.

#### Gaussian filter

Gaussian filtering was a method of weighted average of the values of the data sequence. Each data point was filtered by calculating a weighted average with other values within the field. This process is based on a Gaussian function expressed in Eq. (12), where μ represents the mean value of the Gaussian function, indicating the position of the center point. Since each calculation was based on the position of the current calculation point as the origin, the value of μ was 0.

12 $$ g = \frac{1}{{\sigma \sqrt {2\pi } }}e^{{ - \frac{{\left( {v - \mu } \right)^{2} }}{{2\sigma^{2} }}}} $$

where $v$ is the data that needs to be processed by Gaussian filtering, that is, the initial speed calculated by the distance between the center of the palm and the LeapMotion sensor in the y-axis direction; $\mu$ is the mean value of the Gaussian function, which was taken as 0; $\sigma$ is the standard deviation of the Gaussian function; $g$ is the filtered data of data $v$.

Gaussian filter is characterized by its shape as a bell curve. Within this curve, σ denotes the standard deviation of the Gaussian function, symbolizing the smoothing and denoising impact of Gaussian filtering on the data. When σ is larger, the peak of the curve is smaller, the graph is gentler, and the weight of the data on both sides is greater, that is, the smoothing effect is better; When σ is smaller, the peak of the curve is larger, the change of the graph is larger, and the weight of the data on both sides is smaller, that is, the smoothing effect is worse. Since a palm data was collected every 20 ms in this system, which belonged to high-frequency data, σ needed to take a larger value in this study to increase the weight of domain data. In addition, since the interval time of each data was only 20 ms, taking a larger value of the Gaussian window length would not cause smooth transition and lose the meaning of the original data. After many experiments and tests, this study finally found that the filtering effect was the best when σ was set to 15 and the Gaussian filter window was set to 91.

Statement:

1. We confirm that all experimental protocols were approved by College of Mechanical and Electrical Engineering, Hohai University.
2. We confirm that informed consent to participate was obtained from all subjects and/or their legal guardian(s).
3. We have obtained informed consent from all subjects and/or their legal guardians to publish identifying information/images in online open access publications.
4. We confirm that 0061ll methods were carried out in accordance with relevant guidelines and regulations.

## Experiment and result analysis

### Experiment setup

**Figure 8:**
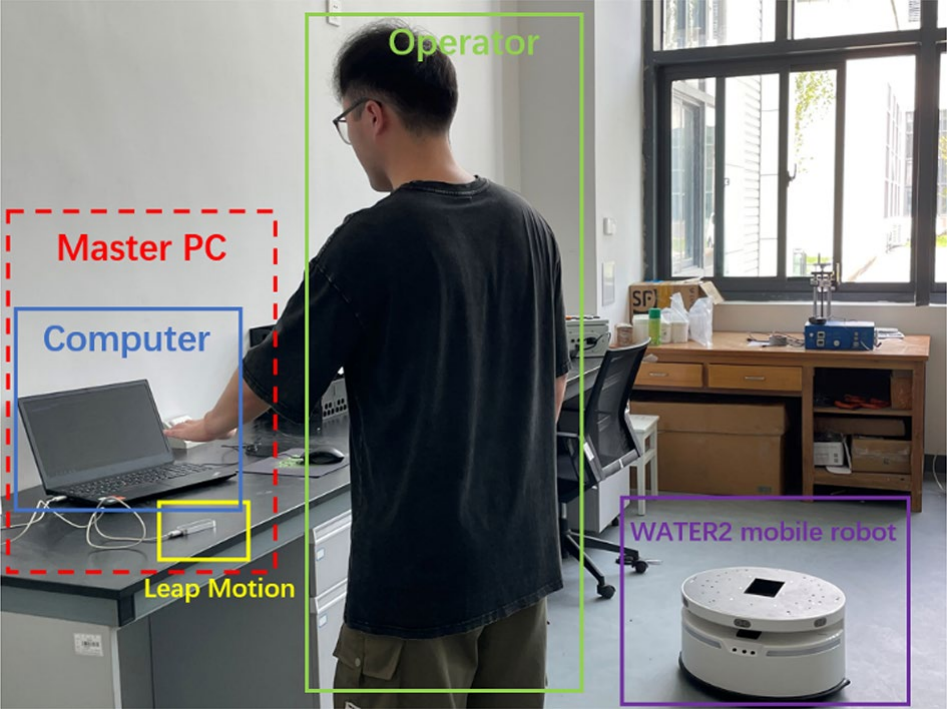
Experiment platform.

We had conducted some experiments to verify the effectiveness of our developed control system, the experimental platforms included: a laptop with windows 10 operating system, pycharm compiler and LeapMotion python SDK V3, LeapMotion sensor and WATER2 mobile robot. Since the recognition accuracy of the LeapMotion sensor is higher in a well-lit environment, the experimental environment of this study was an indoor environment with sufficient lighting. The operator sat in front of the computer to which the LeapMotion sensor and the mobile robot had been connected, and placed his left hand directly above the LeapMotion sensor. After completing the above steps, the experimental platform was shown in Fig. 8, and we would run the control system we developed for experiments.

### Control system verification and analysis

**Figure 9:**
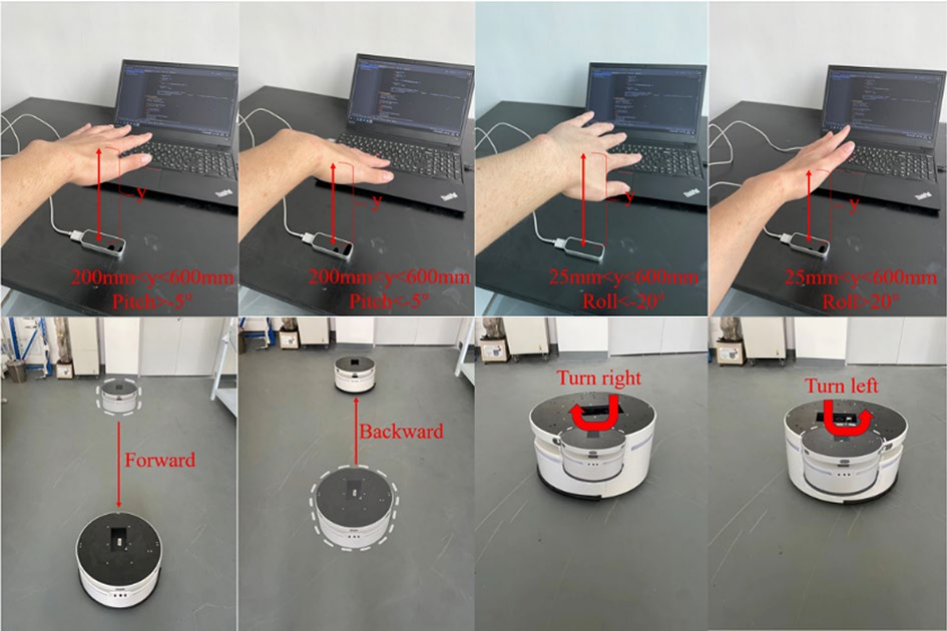
Experimental results.

In order to verify the effectiveness of the designed control system, we designed the following experiments: First, after we placed our hand on the LeapMotion device that had been connected to the computer, we sequentially moved the palm up and down, moved the palm inwards up and down, flipped palm to the right and flipped palm to the left. The corresponding actions of the mobile robot were forward by changing speed, backward by changing speed, turning right and turning left. Figure 9 showed the experimental results. The faded area outlined by the white dotted circle in the figure was the initial position of the mobile robot, and the area that was not faded was the end position of the mobile robot. Based on the final experimental results, we concluded that the mobile robot can quickly respond to gestures and make corresponding actions, which showed that the control system we developed was efficient and practical.

### Time delay and error analysis

In order to study the accuracy and time delay of the designed gesture recognition system, 50 experiments on gesture-controlled the mobile robot were conducted. The number of recognition errors during 50 gestures controlling the mobile robot and the real-time time sent by the computer to the mobile robot were recorded. In addition, the real-time time returned to the computer by the mobile robot were also recorded. The data of 50 experiments were shown in Table 1.

**Table 1:** Experimental data of time delay and error.

Time delay/ms for 50 experiments	168	160	182	166	179	180	172	170	158	166
	169	171	153	169	159	162	167	155	163	188
	177	183	161	170	155	190	169	170	143	149
	165	179	188	172	147	152	159	185	177	171
	192	164	169	159	173	150	187	168	162	160
Number of recognition errors	4									
Average time delay/ms	168.06									
Error rate	0.08									

It could be concluded from Table 1. that a total of four recognition error occurred in 50 experiments, and the error rate was around 0.08. The response delay of mobile robots was between 140 and 200 ms. The average response delay of 50 experiments was 168.06 ms. This delay was short. Therefore, it could be seen that the designed gesture control system had good real-time performance, the program could quickly recognize gesture information, and the mobile robot could quickly make corresponding actions.

**Figure 10:**
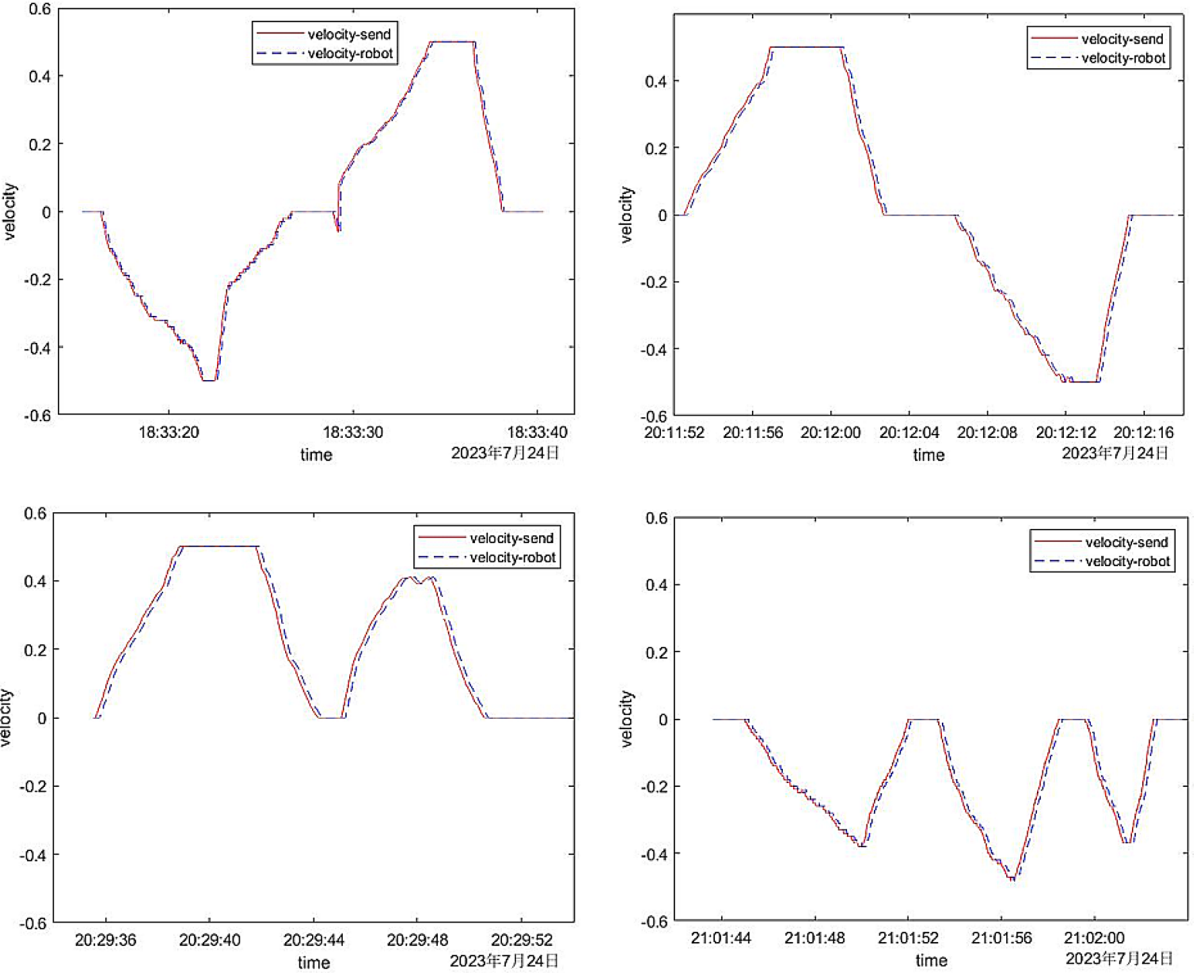
The comparison between the linear speed sent by the computer and the actual linear speed of the robot.

**Figure 11:**
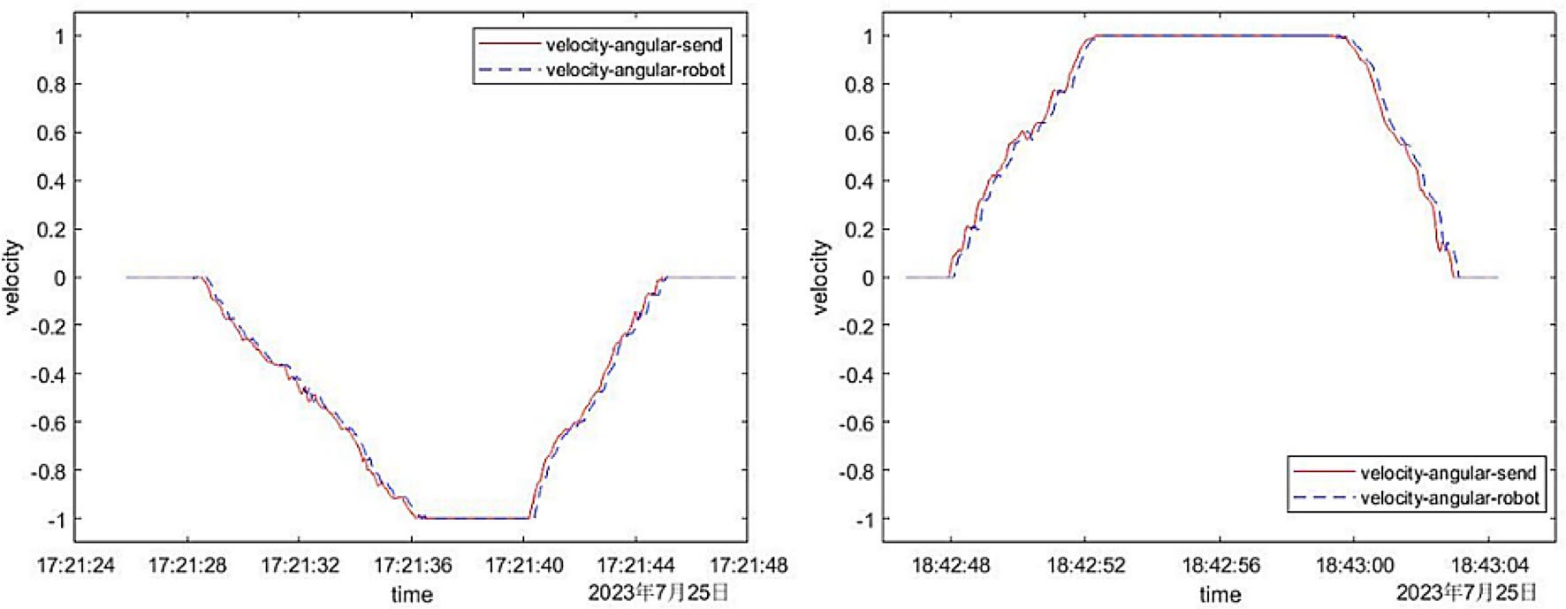
Comparison of the angular velocity sent by the computer and the actual angular velocity of the robot.

To delve deeper into the time delay, precision, and stability of the designed gesture recognition system, we conducted an extensive series of research experiments. The experimental data of six typical control forms were plotted, four of which were experimental data of linear velocity, and two were on angular velocity, as shown in Figs. 10 and 11.

In Fig. 10, the horizontal axis was time, and the vertical axis was linear velocity. The four movement modes of the mobile robot were: (1) first change speed to move backward and then change speed to move forward; (2) first change speed to move forward and then change speed to move backward; (3) change speed forward; (4) change speed backward. The red solid line was the linear velocity sent by the computer to the mobile robot, and the blue dotted line was the actual linear velocity returned by the mobile robot. It could be seen from Fig. 10. that the stability of the mobile robot during the control process was relatively good, and there were no excessive mutations and gesture recognition errors in terms of speed; In addition, in terms of accuracy, the actual linear speed of the mobile robot was the same as the linear speed sent by the computer to the mobile robot, and the speed accuracy was high. However, it was not difficult to find from the figure that the dotted line was shifted a little to the right compared to the solid line. This was the time delay tested above. The time delays are all between 140 and 200 ms, which was consistent with the results of the above test. It could be seen that the time accuracy was also quite high.

In Fig. 11, the horizontal axis was time, and the vertical axis was angular velocity. The two movement modes of the mobile robot were: (1) turn right with variable speed; (2) turn left with variable speed; The red solid line was the angular velocity sent by the computer to the mobile robot, and the blue dashed line was the actual angular velocity returned by the mobile robot. It could be seen from the figure that the response accuracy and response time delay of the angular velocity of the mobile robot were almost the same as the response accuracy and response time delay of the linear velocity above. They all had high accuracy and short time delay. Moreover, there was no unreasonable mutation in the figure, which proved that the stability of the mobile robot during the control process was also good.

### Denoising optimization experiment

During the test, we found that our palms inevitably shook unintentionally. In order to further enhance the robustness and stability of the designed control system, we performed a smooth denoising effect on the velocity array by using exponentially weighted moving average filtering and Gaussian filtering.

#### Effect analysis of exponentially weighted moving average filtering

**Figure 12:**
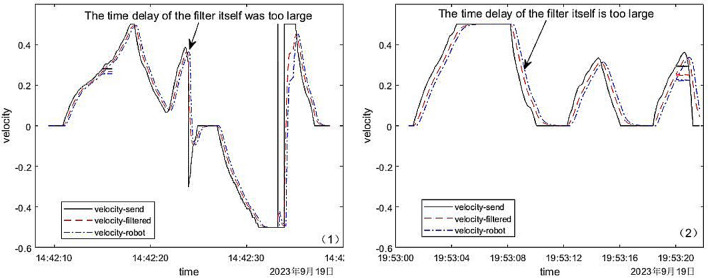
Linear velocity comparison chart before and after exponentially weighted moving average filtering.

We conducted two experiments using exponentially weighted moving average filtering to smooth and denoise the initial velocity. The linear speed before denoising, the linear speed after denoising and the real-time linear speed returned by the mobile robot were compared. The comparison results were shown in Fig. 12.

*Result discussion* The black solid line in Fig. 12 was the original linear velocity, the red dotted line was the linear velocity after exponential weighted moving average filtering, and the blue dotted line was the linear velocity returned in real time during the movement of the mobile robot. From Fig. 12, we find that the red dotted line moved back a certain distance relative to the black solid line, which was the time delay of the exponentially weighted moving average filter itself. Therefore, although the exponentially weighted moving average filter had a smooth denoising effect on the speed of the mobile robot, it itself had a time delay. And judging from the experimental data results, in these two experiments, its own experimental delays were 373 ms and 386 ms respectively, while the system’s own time delays before filtering were only 179 ms and 197 ms respectively. Therefore, the exponentially weighted moving average filter brought a large delay to the control system we design, and was not suitable for the control system we design.

#### Gaussian filter

**Figure 13:**
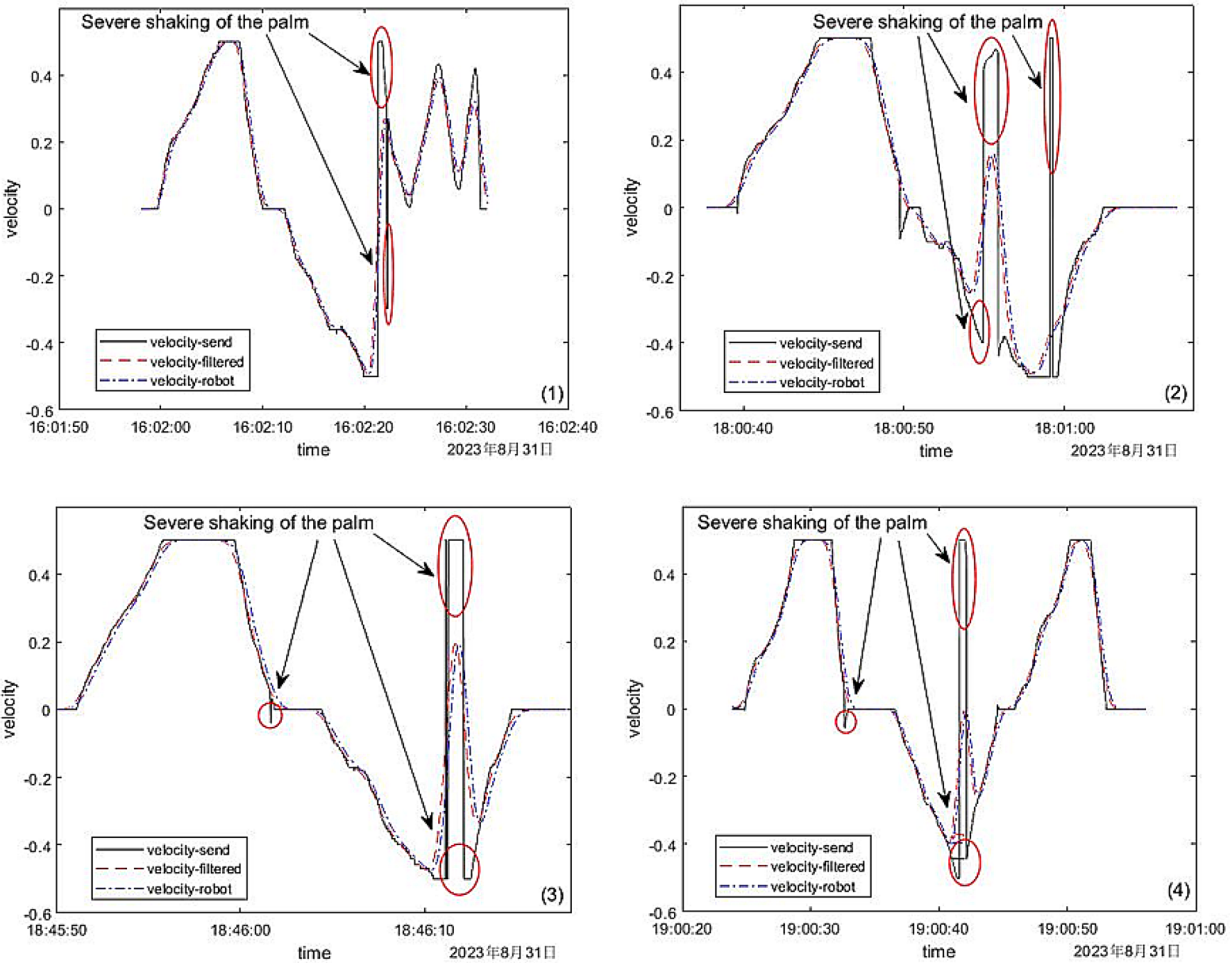
Data comparison of linear velocity before and after filtering.

Then we conducted multiple experiments in which palms trembled, and conducted drawing comparison analysis on four of the experiments. In these four experiments, we used Gaussian filtering to smooth and denoise the linear velocity obtained during the experiment, and then sent the denoised linear velocity to the mobile robot. Then, the linear speed before denoising, the linear speed after denoising and the real-time linear speed returned by the mobile robot were compared. The comparison results are shown in Fig. 13.

*Result discussion* In Fig. 13, the black solid line was the original linear velocity, the red dotted line was the linear velocity after Gaussian filtering, and the blue dotted line was the linear velocity returned in real time during the movement of the mobile robot. From the comparison between the original speed and the speed after filtering, it could be concluded that in the four experiments, the linear velocity changed became gentle after smoothing by Gaussian filtering where the palm shaking were circled in Fig. 13. Moreover, the real-time speed change of the mobile robot after receiving the instruction was also stable and gentle, and it could move smoothly and safely during the movement. Therefore, the application of Gaussian filtering effectively avoided the unstable motion of the mobile robot caused by the shaking of the palm during the control process, and greatly improved the stability and robustness of the system developed in this paper.

Finally, six people were selected to experience the control system, and their experience of using it was recorded. The results showed that all six experiencers said it was easy to get started with the control system, with almost no difficulty in operation and high control accuracy.

### Volunteer evaluation

**Figure 14:**
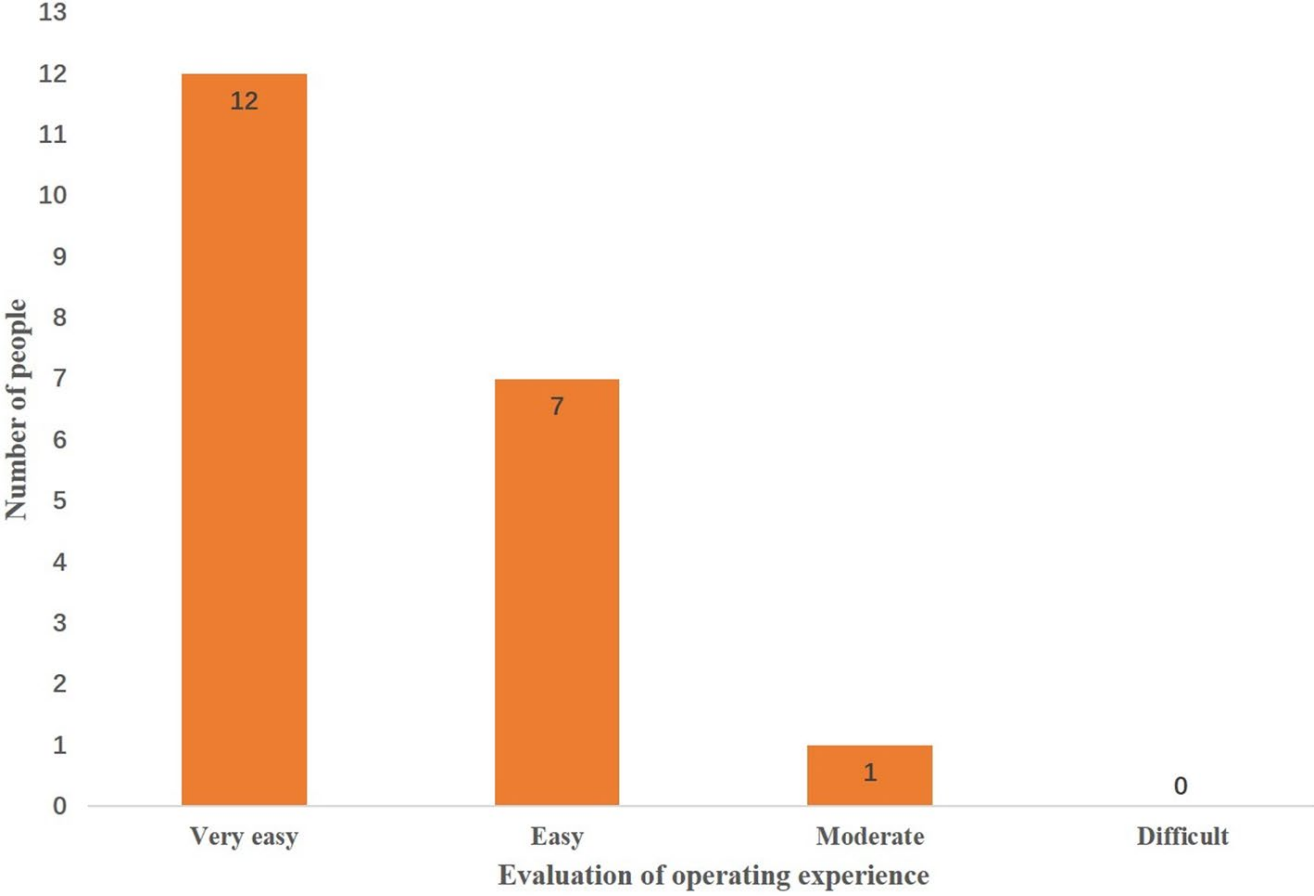
The evaluation of the difficulty of the control process.

In order to ensure that the control system developed in this research is easy to use and suitable for most people, we invited 20 volunteers to experience our control system. We required 20 volunteers to control the mobile robot to move from a fixed location to a fixed end-point through our control system. The two locations are 10 m apart. The total time spent by each volunteer, as shown in Table 2, and the evaluation of the difficulty of the control process, as shown in Fig. 14, were recorded.

**Table 2:** Time spent by each volunteer.

Time spent by each volunteer/s	21	23	25	21	23	26	25	22	21	24
	24	23	25	21	22	23	24	22	25	23
Average time/s	23.15									
Range	5

It could be seen from Table 2 that the average time spent by 20 volunteers was 23.15 s. The difference between the longest time spent and the shortest time was only 5 s. This shown that the control system developed in this research had very little difference in adaptability to different people.

In Fig. 14, 12 out of 20 volunteers thought that the control system developed in our study was very easy to operate, 7 thought it was easy, 1 thought it was moderate, and no one thought it was difficult. Therefore, the gesture-controlled mobile robot system developed in our study is very easy to operate and puts no burden on the operator (Supplementary information).

## Conclusion and future works

In this paper, a gesture-based mobile robot control system was developed using a LeapMotion sensor. The principles of gesture recognition and relevant self-investigated algorithms—GestureMoRo for the association between gestures and mobile robots were studied, which successfully realized the function of gestures to control mobile robot’s locomotion. Moreover, Gaussian filter algorithm was integrated into the designed control system to achieve smooth denoising of the linear velocity of the mobile robot and avoid unstable motion of the mobile robot caused by hand shaking. After many experiments and verifications, the system had been proved that it had the advantages of high precision, good stability and flexible operation. However, it also had the disadvantage of 140–200 ms delay. Moreover, after the linear velocity was smoothed by Gaussian filtering, the robustness and stability of the mobile robot’s locomotion were significantly improved. The original intention of developing this system is to make it easy for people to use in daily life, so the gestures designed are relatively simple and natural. The system can be used for wheelchair control for disabled people, industrial transportation, etc. In future work, shortening the system’s latency and improving the system’s gesture recognition accuracy will be solved. It may also be combined with a robotic arm to apply the operator’s right hand to the system to achieve more control possibilities. Furthermore, the method of detecting the user’s intention will be tried to be applied in this control algorithm, so that the mobile robot can move according to the operator’s intention.

### Supplementary Information

Supplementary Video S1.

Supplementary Legends.

## Data Availability

The datasets used and/or analysed during the current study available from the corresponding author on reasonable request.
