# Supplementary material for: GestureMoRo: an algorithm for autonomous mobile robot teleoperation based on gesture recognition
Source: Sci Rep. 2024 Mar 14;14:6199. doi: 10.1038/s41598-024-54488-w (PMC10940285; doi:10.1038/s41598-024-54488-w)
Supplement: Supplementary file 2 — Supplementary Legends. [file 41598_2024_54488_MOESM2_ESM.doc]

**Video Title:** Gestures control a mobile robot in real time.

**Video Legend:** This supplementary video is mainly used to demonstrate an experimental process of remotely controlling a mobile robot through gestures. By controlling the pitch angle of the palm in the front and rear directions and the roll angle in the left and right directions, the mobile robot can be controlled to move forward, backward, turn left, and turn right. And control the speed of the mobile robot by controlling the distance between the palm and the sensor.
